# Neonatal Dengue Infection Mimicking Late-Onset Neonatal Sepsis in Endemic Regions: A Case Report Series and a Proposed Diagnostic Framework for Antimicrobial Stewardship

**DOI:** 10.3390/microorganisms14091969

**Published:** 2026-09-07

**Authors:** Andrés Arias-Sanchez, Camilo Acosta-Parada, Yaimy Valencia, María Paula Guerrero, Catalina Jaramillo

**Affiliations:** 1 Department of Pediatric Infectious Diseases, Hospital Universitario Erasmo Meoz, Cúcuta 540005, Colombia; felipe_arias4@hotmail.com; 2 Clinical Research Group, Infectoped S.A.S., Cúcuta 540005, Colombia; yaimyvalencia.med@gmail.com (Y.V.); mpguerrerog@unal.edu.co (M.P.G.); 3 Department of Pediatric Infectious Diseases, Sie Salud, Cúcuta 540005, Colombia; catica79@gmail.com

**Keywords:** neonatal dengue, late-onset neonatal sepsis, arboviral infections, antimicrobial stewardship, dengue-endemic regions, neonatal infection, clinical decision framework

## Abstract

Neonatal dengue infection may mimic late-onset neonatal sepsis (LOS) in endemic regions, leading to misdiagnosis and unnecessary antibiotic exposure. We describe a case series of neonates admitted to a tertiary referral hospital in a dengue-endemic area with suspected LOS who underwent standard sepsis evaluation, including blood cultures, inflammatory markers, and clinical assessment, and were subsequently tested for dengue infection by anti-dengue virus IgM using ELFA methodology on the VIDAS^®^ platform. All patients presented with fever and hematologic abnormalities, particularly thrombocytopenia and/or leukopenia, while blood and urine cultures remained negative and procalcitonin levels were low or normal. Dengue IgM was positive in all cases, supporting the diagnosis of neonatal dengue infection. After diagnostic confirmation, antibiotics were discontinued when applicable, and all neonates had favorable clinical outcomes without complications at discharge. These findings highlight dengue as a potentially underrecognized differential diagnosis of LOS in endemic settings and suggest that arboviral testing may be considered in the diagnostic evaluation of neonates with suspected sepsis, particularly when cultures are negative and inflammatory markers are not suggestive of bacterial infection. Early recognition may help reduce unnecessary antimicrobial exposure and could support antimicrobial stewardship efforts.

## 1. Introduction

Late-onset neonatal sepsis (LOS) continues to represent a major contributor to neonatal morbidity and mortality globally [1]. In endemic settings, the clinical approach frequently involves early initiation of broad-spectrum antibiotics due to the high risk of bacterial infection and the non-specific clinical presentation in this population. However, this approach may inadvertently contribute to antimicrobial overuse, microbiome disruption, and selection of resistant organisms [1,2,3]. Bacterial infections have classically been regarded as the main cause of this condition in neonates, with the urinary tract, bloodstream, lungs, and central nervous system usually considered the most relevant potential sources. This clinical approach also increases healthcare use because it commonly leads to hospital admission, additional diagnostic testing, and antimicrobial treatment [4,5]. This approach also increases healthcare costs due to hospital admissions, diagnostic testing, and antimicrobial use [6].

In dengue-endemic regions, the epidemiological burden of arboviral infections represents an additional challenge in the evaluation of febrile illnesses, including neonatal populations [7,8]. Although neonatal dengue has been described, it remains underrecognized, particularly in cases without a clear history of maternal infection or vertical transmission [9]. Early postnatal exposure to *Aedes* vectors creates a plausible pathway for infection shortly after birth, yet this mechanism is often overlooked in routine clinical practice. As a result, neonates presenting with non-specific signs such as fever and hematologic abnormalities may be managed under the assumption of bacterial sepsis, leading to potential misclassification and unnecessary antimicrobial use.

Dengue virus (DENV), the most prevalent arbovirus globally, is rarely considered in neonates outside the context of vertical transmission. Nevertheless, exposure to *Aedes* mosquitoes begins immediately after birth in endemic settings, making postnatal infection biologically plausible. The clinical and hematologic overlap between dengue infection and bacterial sepsis—including thrombocytopenia, leukopenia, and fever—poses a diagnostic challenge [10,11,12]. Currently, no structured diagnostic approach integrates arboviral testing into the evaluation of LOS in endemic areas. This gap may result in underdiagnosis of dengue and unnecessary antibiotic exposure.

In this context, we describe a series of neonates with neonatal dengue infection presenting as suspected late-onset sepsis and propose a conceptual diagnostic approach designed to improve early recognition and optimize clinical management in dengue-endemic settings.

Neonatal dengue diagnosis can be established through the detection of IgM antibodies, NS1 antigen (NS1-Ag), or molecular assays such as polymerase chain reaction (PCR). In this case series, DENV infection was supported by the detection of IgM antibodies using the VIDAS^®^ platform (BioMérieux^®^, Marcy-l’Étoile, France) with ELFA methodology. This approach was considered a useful diagnostic approach in this population; however, potential cross-reactivity with other flaviviruses should be taken into account.

## 2. Clinical Case

Patient 1: A term neonate was admitted at 21 days of life with a 2-day history of unquantified fever and no additional symptoms. There was no relevant perinatal history, and the mother had remained asymptomatic during the 15 days prior to delivery. Physical examination on admission was unremarkable. Initial laboratory tests (Table 1) showed thrombocytopenia (69,000/µL), while C-reactive protein and procalcitonin levels were within normal ranges. Blood and urine cultures were obtained as part of the sepsis workup, and empirical antibiotic therapy with ampicillin plus amikacin was initiated. On the second day of hospitalization, the infectious diseases team evaluated the patient. As blood and urine cultures remained negative after 24 h and inflammatory markers were not suggestive of bacterial infection, dengue IgM testing was requested and returned positive. Antibiotic therapy was discontinued, and supportive intravenous fluids were provided according to clinical response. The patient was discharged on the sixth hospital day without complications.

Patient 2: A term neonate was admitted at 12 days of life with a one-day history of fever and no other relevant past medical history. Admission laboratory tests showed leukocyte count of 4.97 × 10^3^/µL, thrombocytopenia (platelets 91 × 10^3^/µL), and elevated C-reactive protein (12 mg/dL) (Table 1). Given suspected late-onset neonatal sepsis, empirical antibiotic therapy with cefepime plus amikacin was initiated. An evaluation by the Infectious Diseases team was requested. During clinical assessment, it was noted that the patient’s mother had been hospitalized before delivery with a three-day history of fever, myalgias, arthralgias, and abdominal pain. Follow-up laboratory tests showed leukopenia, persistent thrombocytopenia, and elevated transaminase levels. Based on these findings, dengue was considered in the differential diagnosis, and dengue IgM testing returned positive. Dengue with warning signs was diagnosed, antimicrobial therapy was discontinued, and the patient was discharged on the eighth day of hospitalization without complications.

Patient 3: A term newborn with low birth weight without any other relevant past history. Admitted at 4th day of life due to feeding problems, basic studies and serial cultures (blood, urine) were taken. Blood count showed polycythemia; pediatrician indicated proper management. Follow-up laboratory tests showed thrombocytopenia (119,000/mL). The patient was subsequently evaluated by the infectious diseases team, and anti-dengue IgM testing yielded a positive result. Neonatal dengue was diagnosed, and supportive management was initiated. No antimicrobial therapy was required. Although bacterial cultures were negative, the possibility of culture-negative or partially treated neonatal sepsis cannot be completely excluded. However, the clinical evolution, laboratory findings, and response to management were more consistent with a non-bacterial etiology.

## 3. Discussion

Cúcuta, Norte de Santander, is a recognized dengue-endemic region in northeastern Colombia, characterized by sustained transmission and recurrent epidemic periods driven by favorable climatic and vector conditions. This epidemiological context increases the likelihood of early-life exposure, supports consideration of dengue in the differential diagnosis of febrile neonates.

Dengue is infrequently recognized in the neonatal population, resulting in a low level of clinical suspicion, particularly outside the context of vertical transmission. In cases of LOS, the absence of specific diagnostic guidelines further contributes to its under-recognition in this age group [13,14].

Previous reports have described neonatal dengue cases, including small case series from Mexico and other settings. However, most of these studies focus on vertically transmitted infections rather than postnatal acquisition [15,16].

This study demonstrates the presence of neonatal dengue not related to vertical transmission as a differential diagnosis in the approach to late neonatal sepsis in endemic countries in the neonatal population in whom a different viral, bacterial or parasitic etiology cannot be demonstrated, with adequate clinical response, rapid suspension of antibiotics and early discharge. All neonatal dengue diagnoses achieved in our experience were diagnosed with positive IgM, which was considered diagnostic in this population since IgM does not cross the placenta [17]. Only IgM was measured based on the lack of placental transfer. However, NS1-Ag detection in early postnatal infections serves as a complementary tool. Both IgM and NS1-Ag would function as confirmatory tests in the neonatal period. Evidence suggests that IgM levels can begin to rise from day 4 of infection onward in neonatal cases, supporting its role as a confirmatory tool in the early neonatal period [15].

The availability of molecular testing and NS1 antigen detection could substantially improve early identification of dengue infection in neonates presenting with suspected sepsis in endemic settings. Incorporating these diagnostic tools into the initial evaluation may facilitate earlier recognition of viral etiology, allowing timely discontinuation of antibiotic therapy and reducing unnecessary antimicrobial exposure and its associated risks.

In our case series, antibiotic treatment was discontinued in all cases following confirmation of dengue. Notably, one of the neonates was followed up in an outpatient setting, with elevation of quantitative IgG titers confirmed by the same platform (VIDAS 3^®^) ELISA/ELFA performed 14 days after the initial measurement, further supporting the diagnosis. This approach supports the inclusion of dengue in the differential diagnosis in endemic settings, particularly to improve clinical management and minimize unnecessary antibiotic use. In all cases, blood cultures were obtained prior to antimicrobial administration using institutional neonatal sampling protocols with adequate age-adjusted blood volumes. All cultures remained negative. In addition, although culture-negative neonatal sepsis remains a recognized clinical possibility, the combination of persistently negative cultures, early discontinuation of antimicrobial therapy, and absence of clinical deterioration after antibiotic withdrawal made active bacterial sepsis less likely in our patients.

Early discontinuation of antimicrobial therapy may reduce its associated consequences, including alterations in the microbiota, which have been linked to long-term conditions such as obesity, diabetes, and asthma, as well as changes in immune function [18].

Based on these findings, we propose a conceptual diagnostic approach for late-onset neonatal sepsis in dengue-endemic regions that incorporates arboviral infections into the differential diagnosis. In this context, it is important to consider that IgA, IgM, and IgE antibodies do not cross the placental barrier, making them useful markers of active infection in neonates. The addition of NS1 antigen detection and dengue PCR, depending on local availability, may further support diagnostic confirmation [17,18,19].

The increasing circulation of arboviruses underscores the need to expand current diagnostic approaches in endemic areas. Our proposal aims to integrate not only dengue but also other relevant arboviruses, including chikungunya and Zika, as part of a broader differential diagnosis in neonatal settings [20,21].

Neonates in endemic regions are exposed to dengue vectors from birth, which places them at risk of acquiring the infection early in life, along with other circulating arboviruses [20,21]. Despite their young age and the inherent vulnerability of their developing immune system, none of the cases in our series developed severe complications, suggesting a potential protective effect in this population. This observation raises the possibility of passive transfer of homotypic antibodies from seropositive mothers to their newborns. Future studies should explore whether maternally derived antibodies, acquired through placental transfer, provide partial or complete protection against dengue during the neonatal period. Such findings could have important implications for early prevention strategies and for the development of clinical and vaccination approaches in high-prevalence settings.

## 4. Conclusions

Dengue represents a major global public health concern, affecting millions of individuals worldwide, including the neonatal population, in whom transmission may occur through routes other than vertical exposure.

In the neonatal period, the clinical and hematologic features of symptomatic dengue may overlap with those observed in bacterial LOS, which complicates the diagnostic approach. In this context, a structured diagnostic framework that integrates common infectious causes with consideration of dengue is essential for appropriate evaluation in endemic regions.

This approach may also be extended to other arboviral infections in neonatal settings.

**Figure 1:**
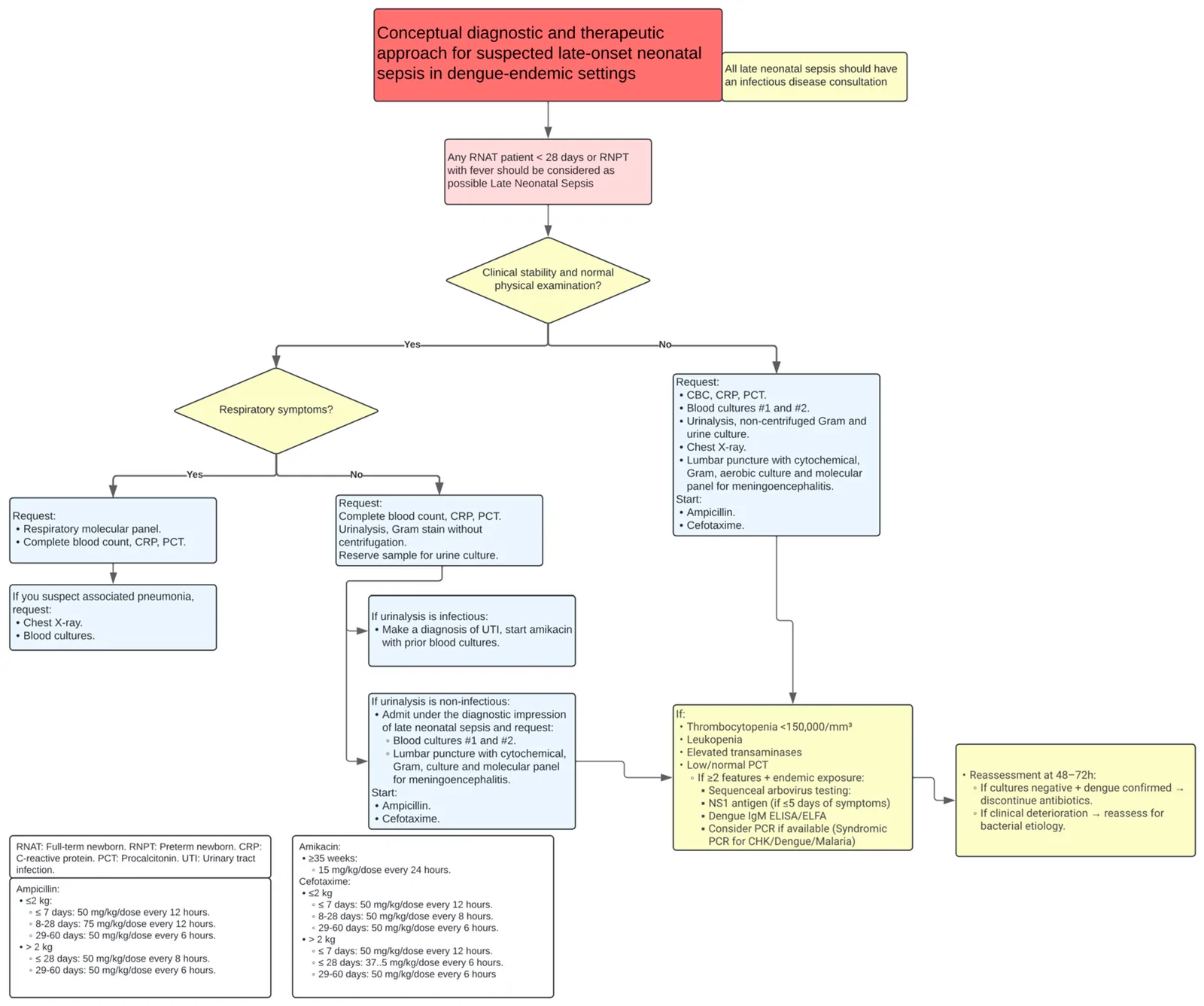
Conceptual diagnostic framework for the evaluation of suspected late-onset neonatal sepsis in dengue-endemic settings. This proposed approach incorporates dengue testing when clinical presentation and laboratory findings suggest a non-bacterial etiology, allowing early recognition of possible arboviral infection and potential discontinuation of unnecessary antibiotic therapy.

Figure 1 proposed conceptual framework for the evaluation of neonates with suspected late-onset neonatal sepsis in dengue-endemic regions.

## Figures and Tables

**Table 1:** Clinical and demographic data of the cases.

Case	Age	Sex	Number of Days of Symptoms	History of Gestational Dengue	Number of Days Since Admission to Infectoped Consultation	Leukocytes Admission 10^3^/µL	Platelets Admission 10^3^/µL	CRP Admission mg/dL	Diagnostic Method	Did You Receive Antibiotics?	Was Antibiotic Therapy Discontinued Early?
1	21 days	Male	1 day	No	2 days	6.61	69	0.1	IgM Dengue	Yes: Ampicillin/Amikacin	Yes
2	12 days	Male	1 day	No	4 days	4.97	91	12	IgM Dengue	Yes: Cefepime/Amikacin	Yes
3	4 days	Female	1 day	No	1 day	18.24	150	0.6	IgM Dengue	No	Not applicable

CRP = C-reactive protein.

## Data Availability

The data supporting the findings of this study are available within the article. Further inquiries can be directed to the corresponding author.
